# Prefrontal and Temporo-Parietal Involvement in Taking Others’ Perspective: TMS Evidence

**DOI:** 10.1155/2008/694632

**Published:** 2008-04-11

**Authors:** Alberto Costa, Sara Torriero, Massimiliano Oliveri, Carlo Caltagirone

**Affiliations:** ^1^I.R.C.C.S. Fondazione Santa Lucia, RomeItaly; ^2^Dipartimento di Psicologia, Università à di Palermo, PalermoItaly; ^3^Clinica Neurologica, Universit `a di Roma “Tor Vergata”, RomeItaly

**Keywords:** Theory of mind, mentalizing, transcranial magnetic stimulation

## Abstract

*Introduction:* Understanding the mental states of others entails a number of cognitive processes known as Theory of Mind (ToM). Behavioural and functional neuroimaging evidence suggests that prefrontal and temporo-parietal cortices are involved in these abilities. The present study was aimed at investigating the role of the dorsolateral prefrontal cortex and temporo-parietal junction in ToM by using a repetitive transcranial magnetic stimulation (rTMS) paradigm.

*Material and Methods:* Eleven healthy subjects participated in the study. The experimental ToM procedure was constituted by false belief and faux-pas written stories. Subjects were evaluated in baseline condition (Sham) and after 1Hz rTMS over the left/right dorsolateral prefrontal cortex and temporo-parietal junction. A score for accuracy and response times were recorded.

*Results:* As regards false beliefs, rTMS over right prefrontal and temporo-parietal areas significantly interfered with response times (*p* < 0.05). The application of rTMS over right/left prefrontal and right temporo-parietal cortices also significantly worsened accuracy in the ability to take the others’ perspective in faux-pas tasks as compared to Sham (*p* ࣘ 0.05 in all cases).

*Conclusions:* The results of the present study are consistent with previous findings supporting the hypothesis that prefrontal and temporo-parietal regions are part of a neural network specifically underpinning the ability to attribute mental states to others.

## 1. Introduction

The attribution of mental states, such as intentions and beliefs, to Others has been referred to as Theory of Mind (ToM) or mentalizing [1]. Recent behavioural and neuroimaging investigations suggested that frontal lobes play a critical role in ToM [2]. Subjects with circumscribed brain damage to prefrontal regions [3–8] have been reported to fail in executing ToM tasks. Coherently, PET and fRMI investigations demonstrated that the lateral and anterior cingulate and the medial/orbitofrontal cortices [9–12] are specifically recruited when subjects perform tasks requiring ToM abilities. However, contrasting data came from other behavioural studies that failed to find a relationship between prefrontal alterations and ToM impairment [13–15]. Differences in executive control and emotional elaboration among the procedures adopted to assess ToM across the studies could in part account for the reported discrepancies. Furthermore, recent studies emphasized the relevance of more posterior cerebral regions in ToM functioning. Samson et al. [16] reported a severe impairment in false belief reasoning in three patients with lesions involving the left temporo-parietal junction (TPj). Moreover, recent fRMI studies demonstrated that the activity of the right TPj was specific to the attribution of mental states [9,11,17].

Summarizing, the reported findings suggest that prefrontal cortex and the TPj might be part of a neural network modulating ToM. The present study was aimed at investigating the effect of inhibitory rTMS over the prefrontal cortex and TPj on the performances of a group of healthy subjects in ToM tasks.

ISSN 0953-4180/08/$17.00 © 2008 – IOS Press and the authors. All rights reserved

## 2. Material and methods

### 2.1. Subjects

Eleven healthy right-handed subjects participated in the study (6 women and 5 men; mean age 22.5 ± 3.0 years). They provided written informed consent for participation in the study, which was approved by the Santa Lucia Foundation's ethical committee.

### 2.2. rTMS protocol

All subjects were evaluated after rTMS over the left and right dorsolateral prefrontal cortex (DLPFC) and over the left and right TPj and at baseline (i.e. Sham), in five different experimental sessions. The order of experimental conditions was randomized among subjects. The sites of stimulation were located using a neuronavigation system (Softaxic) (Fig. 1) and the coil was applied tangentially on the target scalp site, with the handle pointing posteriorly and angled at about 45 ° to the midline. rTMS was delivered by means of a MagStim rapid magnetic stimulator, using a figure-of-eight coil (70 mm in diameter), applied at 1Hz frequency for 15 minutes, at 90% of the motor threshold intensity. As for Sham, the coil was applied on one of the four target sites randomly among subjects and the information given to the subject did not differ from that given in the other experimental conditions.

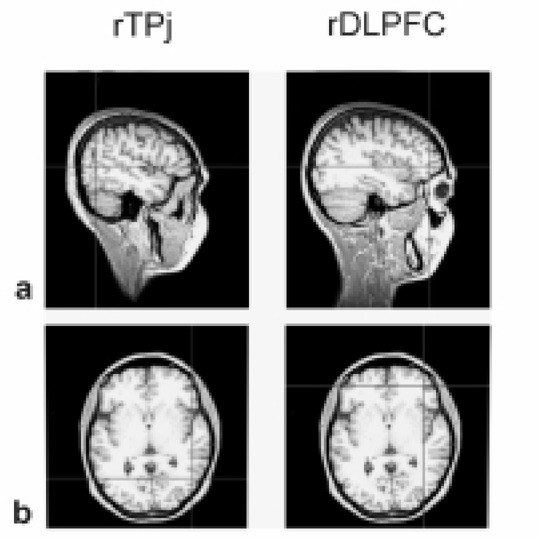

Fig. 1. Anatomical localization of rTMS sites over RMN of a representative subject. Sagittal (**a**) and axial (**b**) sections are reported for both right temporo-parietal junction (rTPJ; Tailarach coordinates: $x = 48$ $y = -55$ $z = 26$) and right dorsolateral prefrontal cortex (rDLPFC; Tailarach coordinates: $x = 38$ $y = 45$ $z = 26$).

### 2.3. Experimental procedure

Based on previously adopted tasks [18,19], we composed 50 written short stories followed by a series of questions. The stories were composed of 20 *first-order false belief* tasks, 20 *Recognition of Faux Pas* tasks and 10 control stories. Five blocks were constructed, each presenting 4 false belief, 4 faux-pas and 2 control stories. The assignment of blocks to the experimental conditions was randomized among subjects. Participants were required to read the stories that appeared on a computer screen and then to answer to three questions by choosing among different alternatives.

*False Belief tasks*: these stories involved one person putting an object somewhere; then the person leaves and a second person moves the object while the other is away. The questions were related to the inference that someone had a mistaken belief (question 1), to the examination of reality (question 2) and to memory accuracy (question 3). *Faux Pas tasks*: in these stories a prior event that occurred between two persons is described. Subsequently the two characters meet each other and one, forgetting the prior occurred event, says something awkward which could offend the other person. The questions required the subjects to detect the faux pas (question 1), to understand the mental state of the listener (question 2) and to understand the mental state of the speaker (question 3). These tasks also included control stories in which no fax pas occurred between the characters.

For all stories, accuracy and response times have been recorded separately for each question. A score of 1 was assigned to correct answers while a score of 0 was given to each wrong answer.

Accuracies after Sham and after rTMS over left/right DLPFC and over left/ right TPj were compared by means of the non-parametric Friedman ANOVA test, separately for each question. The effect of rTMS over DLPFC and TPj on response times to correct answers were evaluated by means of ANOVAs for repeated measures, separately for left and right hemispheres, considering Condition (Sham vs. DLPFC vs. left TPj) and Question as within factors. Tukey HSD tests were performed when required.

## 3. Results

In Table 1 average accuracy and response times for false belief and faux-pas tasks are reported.

Table 1
Average accuracy and response times obtained by subjects in theory of mind tasks

| | Sham | Left rTMS | | Right rTMS | |
|---|---|---|---|---|---|
| | | DLPFC | TPj | DLPFC | TPj |
| False belief stories | | | Mean ± SD | | |
| *Time reading* | 21121 ± 4781 | 21458 ± 5451 | 20019 ± 2995 | 21721 ± 6235 | 21668 ± 5560 |
| *Accuracy* | | | | | |
| Inferring false belief | 3.8 ± 0.4 | 3.8 ± 0.4 | 3.7 ± 0.5 | 3.7 ± 0.4 | 3.5 ± 0.5 |
| Reality exam | 3.6 ± 0.5 | 3.6 ± 0.7 | 3.7 ± 0.5 | 3.6 ± 0.5 | 3.4 ± 0.7 |
| Memory exam | 3.7 ± 0.5 | 3.7 ± 0.5 | 3.8 ± 0.4 | 3.9 ± 0.3 | 3.5 ± 0.7 |
| *Response times* | | | | | |
| Inferring false belief | 5947 ± 1827 | 5546 ± 841.3 | 5773 ± 1463 | 7446 ± 1596 | 7587 ± 2556 |
| Reality exam | 3530 ± 866 | 3498 ± 669 | 3805 ± 1012 | 3968 ± 1123 | 3686 ± 1179 |
| Memory exam | 4189 ± 1930 | 3414 ± 830 | 3196 ± 452 | 4445 ± 2210 | 4295 ± 2053 |
| Faux-pas stories | | | | | |
| *Time reading* | 22482 ± 5244 | 20779 ± 3424 | 20832 ± 5681 | 21787 ± 7051 | 22694 ± 11266 |
| *Accuracy* | | | | | |
| Faux-pas recognition | 3.4 ± 0.9 | 3.7 ± 0.5 | 3.8 ± 0.4 | 3.9 ± 0.3 | 3.9 ± 0.3 |
| Motivation of the listener | 3.5 ± 0.7 | 3.9 ± 0.3 | 3.8 ± 0.4 | 4.0 ± 0.0 | 3.7 ± 0.5 |
| Motivation of the speaker | 3.4 ± 0.7 | 2.5 ± 1.0 | 3.0 ± 0.8 | 2.8 ± 1.1 | 2.7 ± 0.8 |
| *Response times* | | | | | |
| Faux-pas recognition | 2906 ± 903 | 2941 ± 812 | 2684 ± 623 | 3365 ± 1116 | 2813 ± 1078 |
| Motivation of the listener | 4982 ± 1079 | 4902 ± 1370 | 4993 ± 858 | 5530 ± 1183 | 4517 ± 1254 |
| Motivation of the speaker | 8091 ± 1955 | 7646 ± 2229 | 8059 ± 2558 | 7482 ± 3219 | 7747 ± 2907 |

DLPFC = dorsolateral prefrontal cortex; TPj = temporo-parietal junction. Response times are expressed as ms.

### *3.1. False belief tasks*

#### *3.1.1. Accuracy*

Statistical analyses did not reveal significant differences between the score obtained after Sham and after rTMS over the four cerebral sites across the three questions ($\chi^2$ (df = 1) ange from 0.00. to 0.67; $p > 0.10$).

#### *3.1.2. Response times*

rTMS over left DLPFC and left TPj did not significantly affect response times (F consistently <1.50).

The analyses examining the effect of rTMS over right DLPFC and right TPj revealed a significant effect of Condition (i.e. Sham vs. DLPFC vs. TPj), of Question (F(2,20) = 5.72; $p = 0.010$ and F(2,20) = 20.56; $p < 0.001$, respectively) and of the Condition*Question interaction (F(2,40) = 5.05; $p < 0.01$). Post hoc analyses showed that the rTMS over both right DLPFC and TPj increased average response times in false belief detection compared to Sham ($p < 0.001$ in both cases). No other significant effects were found ($p > 0.80$ in all cases).

#### *3.1.3. Faux pas tasks*

As regards control stories, we did not find any significant difference between the experimental conditions both for accuracy and average response times (all $p > 0.10$).

#### *3.1.4. Accuracy*

Subjects were significantly less accurate in their ability to attribute the mental state to the speaker after rTMS over the left DLPFC ($\chi^2$ (df = 1) = 5.44; $p = 0.019$) than after Sham, but not after rTMS on the left TPJ ($\chi^2$ df = 1) = 0.20; $p > 0.50$).

Subjects also showed poorer performances after rTMS over right TPj ($\chi^2$ (df =1) = 4.50; $p < 0.05$) and tended to have lower scores after rTMS over the right DLPFC ($\chi^2$ (df = 1) = 3.57; $p = 0.058$) as compared to Sham. No other significant effects were found ($p > 0.10$).

#### *3.1.5. Response times*

The comparisons between performances after Sham and after rTMS over both hemispheres did not reveal any significant effect (all $p \geqslant 0.10$).

## 4. Discussion

The results indicate that the DLPFC and the right TPj are specifically involved in ToM. In fact, rTMS over both right DLPFC and right TPj significantly increased subjects' response times on false belief tasks for the question that required recognizing the false belief and then predicting the behaviour of one of the characters in the story. The rTMS over these same brain regions as well as over the DLPFC in the left hemisphere was also found to worsen subjects' accuracy in faux-pas stories. The reported effects are not explained by a general interference of rTMS on attentional or memory abilities, in that no effect on the questions investigating memory accuracy was revealed.

These results are not unexpected based on the above mentioned behavioural and neuroimaging findings on the brain correlates of ToM [4–6,9,11,16,17]. Interestingly, at the level of the frontal lobes, it has been suggested that dorsolateral and ventro-medial prefrontal regions participate in different ways in ToM; the former for its contribution to executive and working memory functions strongly implied in ToM tasks, and the latter being more specifically devoted to comprehending other's mental state [6]. Coherently, an association between performances on executive functioning and on ToM-like tasks has been described in patients with DLPFC damage, but not in subjects suffering from lesions involving more ventral regions of prefrontal cortex [5]. Noteworthy, our data also suggest a bilateral involvement of DLPFC in faux-pas stories but not in false beliefs. Indeed, faux-pas tasks require the elaboration of additional emotional and social variables with respect to false-belief tasks [6]. Therefore, the rTMS interference over DLPFC we found on ToM tasks could strengthen the idea that DLPFC is involved in these tasks as a function of the greater executive control they require in being processed.

Our present findings also document a specific involvement of the right TPj in ToM performances. The role of both right and left TPj in ToM has been consistently evidenced by previous studies [9,12,16]. However, our findings are particularly consistent with the results of a recent fRMI study by Saxe and Wexler [17], in which the response of the right TPj, but not the left TPj, revealed a high specificity to the attribution of mental states. The authors also speculated that right TPj might underpin the attribution of relatively transitive mental states, while the left TPj could play a broader role in the elaboration of social enduring relevant traits.

In conclusion, this is the first study directed at investigating the neural correlates of ToM by using rTMS; our data, although preliminary, are consistent with the hypothesis that the DLPFC and the right TPj are part of a distributed neural network underpinning the ability to attribute mental states. The same data also indicate that rTMS may be a promising technique to investigate these processes.
